# Vitamin A Deficiency Exacerbates Gut Microbiota Dysbiosis and Cognitive Deficits in Amyloid Precursor Protein/Presenilin 1 Transgenic Mice

**DOI:** 10.3389/fnagi.2021.753351

**Published:** 2021-11-01

**Authors:** Bo-Wen Chen, Kai-Wen Zhang, Si-Jia Chen, Chun Yang, Peng-Gao Li

**Affiliations:** ^1^School of Public Health, Capital Medical University, Beijing, China; ^2^Beijing Key Laboratory of Environmental Toxicology, Beijing, China; ^3^Beijing Key Laboratory of Clinical Epidemiology, Beijing, China

**Keywords:** vitamin A deficiency, Alzheimer’s disease, gut microbiota dysbiosis, cognitive deficits, APP/PS1 transgenic mice

## Abstract

Vitamin A deficiency (VAD) plays an essential role in the pathogenesis of Alzheimer’s disease (AD). However, the specific mechanism by which VAD aggravates cognitive impairment is still unknown. At the intersection of microbiology and neuroscience, the gut-brain axis is undoubtedly contributing to the formation and function of neurological systems, but most of the previous studies have ignored the influence of gut microbiota on the cognitive function in VAD. Therefore, we assessed the effect of VAD on AD pathology and the decline of cognitive function in AD model mice and determined the role played by the intestinal microbiota in the process. Twenty 8-week-old male C57BL/6J amyloid precursor protein/presenilin 1 (APP/PS1) transgenic mice were randomly assigned to either a vitamin A normal (VAN) or VAD diet for 45 weeks. Our results show that VAD aggravated the behavioral learning and memory deficits, reduced the retinol concentration in the liver and the serum, decreased the transcription of vitamin A (VA)-related receptors and VA-related enzymes in the cortex, increased amyloid-β peptides (Aβ40 and Aβ42) in the brain and gut, upregulate the translation of beta-site APP-cleaving enzyme 1 (BACE1) and phosphorylated Tau in the cortex, and downregulate the expression of brain-derived neurotrophic factor (BDNF) and γ-aminobutyric acid (GABA) receptors in the cortex. In addition, VAD altered the composition and functionality of the fecal microbiota as exemplified by a decreased abundance of *Lactobacillus* and significantly different α- and β-diversity. Of note, the functional metagenomic prediction (PICRUSt analysis) indicated that GABAergic synapse and retinol metabolism decreased remarkably after VAD intervention, which was in line with the decreased expression of GABA receptors and the decreased liver and serum retinol. In summary, the present study provided valuable facts that VAD exacerbated the morphological, histopathological, molecular biological, microbiological, and behavioral impairment in the APP/PS1 transgenic mice, and the intestinal microbiota may play a key mediator role in this mechanism.

## Introduction

Alzheimer’s disease (AD) is a devastating neurological disease characterized by a loss of cognitive function and a gradual decline in daily life activities, accompanied by behavioral changes and various neuropsychiatric symptoms (Joe and Ringman, 2019). It is predicted that there will be 131.5 million patients suffering from AD in the world by 2050 (Hsiao et al., 2018). Although the pathological process of AD is extremely complex and has not been fully elucidated so far, it is known that the typical pathological features of AD are the formation and deposition of extracellular β-amyloid (Aβ) and intracellular neurofibrillary tangles formed by the excessive phosphorylation of Tau protein in neurons, causing a series of reactions, such as the release of inflammatory factors, energy metabolism disorders, and oxidative stress in neurons, and eventually, leading to the degeneration and loss of neurons in cerebral cortex and hippocampus (Reitz et al., 2011). Therefore, preventing the formation of these pathological phenomena will be the key to solve this problem.

As an essential micronutrient, vitamin A (VA) and its derivatives are closely related to the development of the central nervous system (CNS) (Malaspina and Michael-Titus, 2008) and are essential for normal learning and memory functions (Sherwin et al., 2012). VA deficiency (VAD) is one of the most significant micronutrient deficiencies that pose a severe threat to the health of patients with AD in many countries (Lopes et al., 2014). It has been found that VA and β-carotene levels in patients with AD are significantly lower than those in normal controls, which may be partly attributed to the alterations in dietary behavior (Lopes et al., 2014).

It is of great significance to assess the effect of VAD on the pathogenesis of AD. As an active metabolite of VA, retinoic acid (RA) binds to the retinoic acid receptors (RARs) on the nuclear membrane and stimulates the transcription of target genes, participating in the regulation of organ formation and development. Nuclear retinoid X receptors (RXRs) areRA receptors activated by the 9-cis RA and potentially other endogenous retinoids in the CNS (Krezel et al., 1999). The RAR-RXR heterodimer could combine with the RA response element (RARE) in the promoter region of the target genes, thereby regulating the AD-related gene expression (Maden, 2007). In addition, retinaldehyde dehydrogenase 1 (RALDH1) has two main functions: one is to increase the synthesis of RA when retinol is less available; the other is to participate in the synthesis of neurotransmitters, such as γ-aminobutyric acid (GABA). Furthermore, RAR dysfunction can lead to Aβ deposition, impairment of the long-term synaptic plasticity, and memory in the brain of the patients with AD, which can be rescued by the VA supplementation (Misner et al., 2001; Nomoto et al., 2012). It was previously observed that VA can modulate the expression of β-amyloid precursor protein (APP) and the α-secretase to reduce the formation of the oligomerization of Aβ40/Aβ42 *in vitro* (Shudo et al., 2009). Moreover, Wang et al. (2015) demonstrated that VA could reduce the expression of beta-site APP-cleaving enzyme 1 (BACE1), which is a crucial enzyme involved in catalyzing the formation of Aβ polypeptide and cleavage of APP (Leng and Edison, 2020).

The gut microbiota is considered as an invisible organ, which plays a mediating role in the bidirectional signal transduction between the gut-brain axis (Petra et al., 2015). The gut-brain axis conducts bidirectional communication between the gut and the brain through the immune system, enteric nervous system, microbial metabolites, and vagus nerve, which ultimately contributes to the formation and function of neurological systems (Doifode et al., 2021). Many specific bacterial genera in the gut, such as *Lactobacillus*, regulate the expression of specific receptors in the brain *via* the vagus nerve (Bravo et al., 2011). Additionally, the intestinal microbiota is known to secrete immunogenic mixtures into the surrounding environment, such as amyloids, lipopolysaccharides, and other microbial exudates, which may regulate the signaling pathways and produce proinflammatory cytokines in the AD pathogenesis (Zhao et al., 2015). Previous research has indicated that the intestinal microbiota composition can be regulated by diet and specific nutrients, resulting in the production or aggregation of amyloid protein in the brain (Scott et al., 2013). Therefore, the gut-brain axis may play an important role in the process of VAD aggravating the cognitive function of AD.

Emerging evidence shows that VAD in the different life stages could impair gut microbiota homeostasis, leading to the imbalance of *Firmicutes* and *Bacteroidetes* in rat colonic mucosal microbiota (Chen et al., 2020). Amit-Romach et al. (2009) illustrated that VAD could change the composition of gut microbiota and damage the integrity of the gastrointestinal mucosal barrier by reducing the abundance of *Lactobacillus* and the total bacterial count in the gastrointestinal tract. In addition, the alterations in the gut microbial community can reduce the level of brain-derived neurotrophic factor (BDNF), which is considered a genuine molecular mediator of functional and morphological synaptic plasticity in the brain (Bercik et al., 2011). Thus, alterations in the function and composition of the intestinal microbiota can help to recognize the new mechanisms that account for the impact of VAD on the pathogenesis of AD.

To date, the specific mechanism of how VA affects the cognitive function of patients with AD has not been clarified. This research aimed to assess the effect of VAD on the cognitive function and pathological mechanism of AD and determine the intestinal microbiota changes driven by VAD in the amyloid precursor protein/presenilin 1 (APP/PS1) transgenic mice. Our study implies that VAD aggravates the decline of learning and memory function of AD, and intestinal microbiota may play an important role in this process.

## Materials and Methods

### Animals and Diet

In this study, 20 specific pathogen-free (SPF) male C57BL/6J APP/PS1 transgenic mice aged 8 weeks, weighing 20 ± 2 g, were purchased from Beijing Huafukang Biotechnology Co., Ltd. (Beijing, China), and were randomly separated into two parallel groups (*n* = 10 per group). They were raised in standard individual ventilated cages with a 12-h dark/light cycle in a room with controlled temperature (23–25°C) and humidity (45–55%). The VAD feed (VA < 120 IU/kg) and VA-normal feed (VAN; 15,000 IU/kg VA) were purchased from Keao Xieli Feed Co., Ltd. (Beijing, China). Both the feeds and drinking water were available to the mice *ad libitum*. Considering that the mouse species has a high VA storage capacity and needs a long time (up to 40 weeks) (Etchamendy et al., 2003) to induce VAD, we conducted a 45-week dietary intervention in the present experiment. After 45 weeks of dietary intervention, we assessed their cognitive deficits with the Morris water maze (MWM) test and the step-down passive avoidance (SDPA) test.

### Reversed-Phase High-Performance Liquid Chromatography

Since the liver is the most important organ for storing VA and the serum retinol is a common indicator of VAD (WHO, 2009), we used reversed-phase high-performance liquid chromatography (RP-HPLC) to determine the concentration of retinol in the liver and the serum. The serum and liver samples were pretreated with acetonitrile to precipitate the protein. The resulting supernatant was added to 1,000 μl hexane, allowing the retinol to be extracted into hexane. The extract was evaporated to dryness under nitrogen, reconstituted in methanol, and then, separated with methanol in an isometric separation on an HPLC system (Agilent Technologies Inc., CA, United States) with a Pursuit XRs 100A C18 (4.6 mm × 250 mm) column. We set the flow rate to 0.8 ml/min and the detection wavelength to 325 nm to achieve a better detection effect. The peaks were identified by comparing the retention times with those of the standard samples.

### Morris Water Maze Test

The spatial learning and memory abilities of mice were assessed by the MWM test, which includes orientation navigation and spatial probe tests. MWM was carried out in a stainless-steel circular pool with a diameter of 1.2 m and a height of 0.5 m. A round table with a diameter of 9 cm was placed in the first quadrant of the pool and placed 1 cm below the surface of the water. We added titanium dioxide to the pool water and mixed it evenly to make it milky white so that the animal cannot recognize the position of the platform in the pool.

The orientation navigation was carried out for 5 consecutive days. The mice were placed into the pool water facing the wall of the pool at different quadrant positions, and the time (the escape latency) and swimming trajectory of the mice from entering the water to finding the round platform within 90 s were recorded with an image automatic acquisition and software analysis system (Xin Ruan Information Technology Co., Ltd., Shanghai, China). Then, the spatial probe test was carried out on the sixth day. After the platform was removed, the mice were allowed to enter the pool in the quadrant opposite to the platform and search for the platform based on memory. The time spent in the target quadrant, the number of times across the platform, and the swimming trajectory were recorded.

### Step-Down Passive Avoidance Test

The state-dependent learning and memory were evaluated by SDPA, which includes training sessions and testing sessions. The SPDA test was performed in a small chamber containing an insulated wooden platform at the corner and a floor covered with electrode strips. During the training session, the mice were put on a wooden platform and received electric shocks as soon as they stepped on the electrode strips on the floor. The number of times the mouse stepped down from the wooden platform (number of errors) was recorded. After 24 h, the testing session was performed. The electrode strips on the floor were no longer energized. The mice were placed on the wooden platform again, and the time of jumping from the wooden platform to the floor (step-down latency) was recorded.

### Hematoxylin-Eosin Staining

The mouse brain tissue samples were stored in 10% formalin solution, dehydrated according to the conventional methods, and then embedded in paraffin. After the sections were deparaffinized with xylene and different ethanol concentrations, they were stained with hematoxylin for 15 min. Afterward, it was soaked in acidified hydrochloric acid ethanol for 5 s and stained with eosin for 4 min. Finally, each section was examined with a microscope slide scanner (3DHISTECH Co., Ltd., Budapest, Hungary).

### Immunohistochemistry

The mouse brain sections were placed in a small box filled with ethylenediamine tetraacetic acid (EDTA) antigen retrieval buffer (pH 9.0) and boiled in a microwave oven for 5 min; then, placed in 3% hydrogen peroxide solution and incubated for 20 min to inactivate the endogenous peroxidase activity. Next, placed the slides in PBS (pH 7.4), and washed them three times with shaking on a decolorizing shaker, 5 min each time. Then, they were incubated overnight with the primary antibodies: anti-Aβ40-42 (1:500, Abcam, Cambridge, United Kingdom) and anti-BDNF (1:500, Abcam, Cambridge, United Kingdom). Covered the tissue with the horseradish peroxidase (HRP)-conjugated secondary antibody (1:2,000, Abcam, Cambridge, United Kingdom) and incubated for 60 min at room temperature in the dark chamber. Finally, it reacted with 3,3′-diaminobenzidine (DAB) solution for 10 min. Similar to the Hematoxylin-Eosin (H&E) sections, immunohistochemistry (IHC) sections are visualized using the Micro Slide Scanner (3DHISTECH Co, Ltd., Budapest, Hungary). Analytical software (Image J) was used to quantify the integral optical density (IOD) of Aβ and BDNF in each image.

### Western Blot

The lysis buffer was prepared by adding 1 mM PMSF, 10 mM NaF, and 1 mM Na_3_VO_3_ to the RIPA buffer (50 mM Tris buffer saline, 0.5% sodium deoxycholate, 1 mM EDTA, 150 mM NaCl, and 1% NP-40). Total proteins from the mouse cortical tissues were extracted using the lysis buffer. The concentrations of the protein in the extracts were determined by using the BCA test kit (Servicebio Technology Co., Ltd., Wuhan, China) according to the instructions from the manufacturer. Then, 30 (μg of protein was added to each lane, and the protein was separated by sodium dodecyl sulfate-polyacrylamide gel electrophoresis (SDS-PAGE), and then transferred to polyvinylidene fluoride (PVDF) membranes (Merck Millipore Co., Ltd., Darmstadt, Germany). After blocking with 5% (w/v) bovine serum albumin (BSA) for 2 h at room temperature, the membranes were incubated with the following primary antibodies overnight at 4°C: anti-BACE1 (1:1,000, Abcam, Cambridge, United Kingdom), anti-Tau (1:1,000, Abcam, Cambridge, United Kingdom), anti-phosphorylated-Tau (phospho S396, 1:1,000, Abcam, Cambridge, United Kingdom), anti-BDNF (1:1,000, Abcam, Cambridge, United Kingdom), anti-GABA_Aα2_ (1:1,000, Abcam, Cambridge, United Kingdom), anti-GABA_B1b_ (1:1,000, Abcam, Cambridge, United Kingdom), and anti-beta actin (1:5,000, Abcam, Cambridge, United Kingdom). Afterward, the PVDF membrane was incubated with the HRP-conjugated secondary antibody (1:5,000, Abcam, Cambridge, United Kingdom) at room temperature for 1 h. After that, the membranes were stripped by the stripping buffer (Beijing Solarbio Science & Technology Co., Ltd., Beijing, China), and reprobed with β-actin antibody as a loading control. Immunoreactive bands were visualized using the ECL Plus Reagent kit (Merck Millipore Co., Ltd., Darmstadt, Germany). The protein bands were quantified by densitometry using the Image System Fusion FX (Vilber Lourmat Co., Ltd., Paris, France) and normalized to β-actin.

### Real-Time Quantitative PCR

Total RNA of the hippocampus and cortex of the APP/PS1 mice was extracted with the TRIzol^®^ reagent (Thermo Fisher Scientific Co., Ltd., MA, United States). The bacterial genomic DNA was extracted from the feces of the mice with the TIANamp Stool DNA Kit (Tiangen Biotech Co., Ltd, Beijing, China). To ensure the purity of RNA, we used a Genomic DNA pollution scavenger (Applygen Biotech Co., Ltd, Beijing, China) to remove the genomic DNA from the RNA extract. RNA and DNA quality and yield were determined by the ratio of OD260/OD280 with an ultraviolet spectrophotometer (NanoDrop R ND-1000, Thermo Fisher Scientific Co., Ltd., MA, United States). Then, 1 μg of the total RNA from each sample was reverse-transcribed using a RevertAid First Strand cDNA Synthesis kit (Thermo Fisher Scientific Co., Ltd., MA, United States) as prescribed by the supplier. Real-time quantitative PCR (RT-qPCR) was conducted with a Real-time PCR Detection System (Bio-Rad Laboratories Co., Ltd., MA, United States) using KAPA SYBR^®^ FAST qPCR Master Mix (2X) Kit (KAPA Biosystems, Co., Ltd., MA, United States) based on the instructions from the manufacturer. The housekeeping gene β-actin served as the reference for standardization in the hippocampus and cortex, while the universal 16S rRNA gene was used as an internal reference in the fecal samples. The target genes and DNA levels relative to internal reference were quantitatively determined by the 2^–ΔΔCT^ method. The sequences of the primers, such as the genus-specific primer for *Lactobacillus* and *Clostridia_UCG-014* used for RT-qPCR, are listed in Table 1.

**TABLE 1:** The sequences of primers used in the present study.

Genes	Forward sequences (5′→3′)	Reverse sequences (5′→3′)
β-Actin	TGCTGTCCCTGTATGCCTCTGG	ACCGCTCGTTGCCAATAGTGATG
RAR-α	AACAACAGCTCAGAACAAC	CGAACTCCACAGTCTTAATG
RAR-β	CTTGGGCCTCTGGGACAAAT	TGGCGAACTCCACGATCTTAAT
RAR-γ	CCCAAGGATGCTGATGAAAATC	CTCCATCTTCAGGGTTATAGCC
RALDH1	ACTGTGTCATCTGCTCTG	TTACTCTGCTGGCTTCTT
RALDH2	ACATCAACAAGGCTCTCA	CCAAACTCACCCATTTCTC
RALDH3	AGAGGGCTGTTCATCAAA	TGCTGTGAGTCCATAGTC
RXR-α	CTCAATGGCGTCCTCAAGGTTCC	TGTCTCGGCAGGTGTAGGTCAG
RXR-β	TGACCTACTCGTGTCGTGATAA	CTGATAGCGACAGTACTGACAG
RXR-γ	GTTGGTGTCCAAGATGAAAGAC	ATACTTCTGCTTGGTATAGGCC
BACE1	CAAGACGACTGTTACAAGTTCG	TCGAAGACGACATAGAAACCTT
CYP26B1	AGCAAGGAACATGGCAAGGAGATG	GTGGCGTAGGCTGCGAAGATC
ADAM10	GAAGGTTTCATCAAGACTCGTG	CTCCAGTCATTTGGTACTTCCT
BDNF	CGACGACATCACTGGCTGACAC	GCTGTGACCCACTCGCTAATACTG
GABA_Aα2_	ACTTACAATGCTTCTGACTCCGTTCA	GCTGGCACCGATTCTCTGTTCA
GABA_B1b_	GGATGTGGAACCTTATTGT1GCTCTCA	GATTGTGGAGTGTGGCGGATGG
Bacteria 16S	GCTCGTGTCGTGAGATGTT	TGTAGCCCAGGTCATAAGG
*Lactobacillus*	CCCTAAAGACTGGGATACCAC	TACGCATCATTGCCTTGG
*Clostridia_UCG-014*	CGCAATGGAGGAAACTCTG	CTACGCACCCTTTACACCC

*RAR-α, retinoic acid receptors α; RAR-β, retinoic acid receptors β; RAR-γ, retinoic acid receptors γ; RALDH1, retinaldehyde dehydrogenase 1; RALDH2, retinaldehyde dehydrogenase 2; RALDH3, retinaldehyde dehydrogenase 3; RXR-α, retinoid X receptors α; RXR-β, retinoid X receptors β; RXR-γ, retinoid X receptors γ; BACE1, beta-site APP-cleaving enzyme 1; CYP26B1, cytochrome P450 family 26 subfamily B member 1; ADAM10, a disintegrin and metalloproteinase 10; BDNF, brain-derived neurotrophic factor; GABA_*A*α2,_ gamma-aminobutyric acid A subunit alpha 2 receptor; GABA_*B1b*_, gamma-aminobutyric acid B subunit 1 beta receptor.*

### Luminex xMAP^®^ Technology

The concentrations of Aβ40 and Aβ42 in the brain and gut of the mice were measured using the Luminex xMAP^®^ technology and a specific multiplex plate, MILLIPLEX^®^ MAP Mouse Amyloid Beta Magnetic Bead Panel kit (Merck Millipore, Co., Ltd., Darmstadt, Germany) following the instructions from the manufacturer. The Luminex xMAP^®^ technology is to covalently cross-link the antibody molecules against different test substances to specifically coded microspheres and then use flow cytometry to detect the corresponding items of each coded microsphere. The multiplex plate was measured using a Luminex 200^®^ analyzer (Luminex, Co., Ltd., TX, United States). The concentrations of Aβ40 and Aβ42 were analyzed using the ELISAcalc software with a five-parameter logistic curve-fitting method.

### Bacterial 16S rRNA Gene Sequencing

As mentioned above, the fecal bacterial genomic DNA is extracted using the TIANamp Stool DNA Kit (Tiangen Biotech Co., Ltd., Beijing, China) according to the instruction from the manufacturer. The library of each sample was generated by PCR amplification using the universal specific primers 338F (5**′**-ACTCCTACGGGAGGCAGCAG-3**′**) and 806R (5**′**-GGACTACHVGGGTWTCTAAT-3**′**) targeted in the variable V3–V4 regions of 16S rRNA (Caporaso et al., 2011). The expected amplicon size is about 468 bp, and then AMPure XP beads (Beckman Coulter, Inc., CA, United States) are used to purify these amplicons to remove the unbound primers or primer dimers. The DNA concentration of the amplified sequence library was determined using the Qubit quantification system (Thermo Fisher Scientific Co., Ltd., MA, United States). Then, the paired-end sequencing was conducted on the purified libraries using the Illumina MiSeq PE 300 platform (Illumina, Inc., CA, United States).

### Bioinformatic Analysis

As previously described (Edgar, 2013), the operational taxonomic units (OTUs) were clustered using UPARSE (version 7.0) with the threshold of 97% sequence similarity. The Ribosomal Database Project (RDP) Bayesian algorithm was conducted to taxonomy up to the genus level using the Greengenes 13_8 reference database with a confidence threshold of 70% (McDonald et al., 2012). After filtering out the nonaligned and chimeric OTUs and singletons, the final OTU table is generated using Quantitative Insights Into Microbial Ecology 2 (QIIME2) (Bolyen et al., 2019). The difference in abundance between the two groups was analyzed by Mann–Whitney U test and Metastats analysis. The intestinal microbiota diversity within a single sample, namely α-diversity, was assessed using the four indices, the Shannon, Chao1, Ace, and Faith’s phylogenetic diversity (PD) indices, which describe the richness and evenness of each sample. Welch’s *t*-test was performed to assess α-diversity between the two groups. To assess the β-diversity, the principal coordinates analysis (PCoA) plots were obtained based on the unweighted UniFrac distances (Lozupone and Knight, 2005), which were visualized using the Emperor 0.9.4 (Vazquez-Baeza et al., 2013). A permutational multivariate ANOVA (PERMANOVA) was performed to quantify the difference in the β-diversity analysis. The linear discriminant analysis (LDA) effect size (LEfSe) analysis was performed to evaluate the differentially abundant taxa and biological relevance across the groups with a score cutoff of 2. Functional potential profiling of the microbial communities was predicted from the Kyoto Encyclopedia of Gene and Genomes (KEGG) database using the phylogenetic investigation of communities by reconstruction of unobserved states (PICRUSt) (Kanehisa et al., 2012). Spearman rank correlation was performed to examine the associations of the liver retinol and the serum retinol with the abundance of the genus by using R (version 4.0.0) (R Core Team, Vienna, Austria).

### Statistical Analysis

Excluding the microbiota bioinformatic analysis mentioned above, the data from other analyses were represented as mean ± SD. A two-tailed, unpaired Student’s *t*-test was used to compare the difference between the two groups by using the SPSS 23.0 software (IBM, NY, United States). The differences were considered statistically significant at *P* < 0.05, and presented by the asterisks as follows: *^∗^P* < 0.05, *^∗∗^P* < 0.01, and *^∗∗∗^P* < 0.001.

## Results

### Vitamin A Deficiency Reduced Liver and Serum Retinol Levels in the Amyloid Precursor Protein/Presenilin 1 Transgenic Mice

**FIGURE 1:**
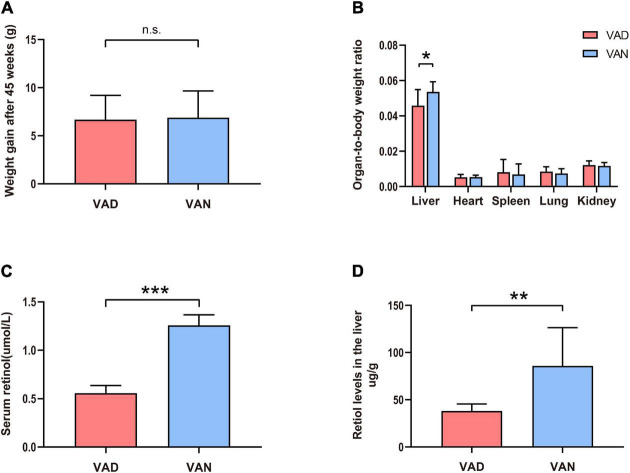
Consumption of a vitamin A deficiency (VAD)-diet for 45 weeks led to reductions in the serum and liver retinol levels. **(A)** Weight gain of the amyloid precursor protein/presenilin 1 (APP/PS1) transgenic mice after 45 weeks on VAD-diet. **(B)** The organ-to-body weight ratio in the APP/PS1 transgenic mice after 45 weeks on the VAD-diet. **(C)** The serum retinol level of the APP/PS1 transgenic mice after 45 weeks on VAD-diet. **(D)** The liver retinol level of the APP/PS1 transgenic mice after 45 weeks on VAD-diet. VAD, vitamin A deficiency diet; VAN, vitamin A normal diet. *n* = 10 per group. ^∗^*P* < 0.05, ^∗∗^*P* < 0.01, ^∗∗∗^*P* < 0.001, and n.s., non-significant.

Compared with the VAN-diet-fed mice, there was no significant decrease in the bodyweight of the VAD-diet-fed mice after 45 weeks of VA deprivation (n.s.; Figure 1A). However, as shown in Figure 1B, the liver-to-body weight ratio was markedly decreased in the VAD group compared with the VAN group (*P* < 0.05). To confirm whether the established mice model was VAD or marginal VAD, we detected the serum retinol (Figure 1C) and found that the serum concentration of VAD-diet-fed mice matched the recommended standards (WHO, 2009) for VAD (≤0.7 μmol/L). Considering that the liver is the primary organ for VA storage in the body, we measured the retinol level in the liver by RP-HPLC and found that it was significantly decreased in the VAD group compared with the VAN group (*P* < 0.01; Figure 1D). The representative HPLC traces of retinol in the liver and serum are shown in Supplementary Figures 1, 2.

### Vitamin A Deficiency Reduced the mRNA Expression of Vitamin A-Related Receptors and Vitamin A-Related Enzymes in the Cortex

**FIGURE 2:**
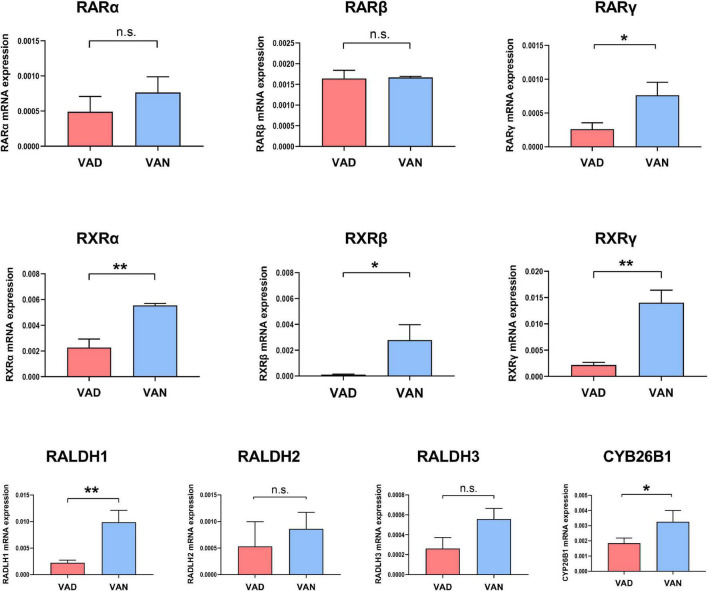
Impact of consumption of the VAD-diet for 45 weeks on the mRNA expression of the vitamin A (VA)-related receptors and VA-related enzymes in the cortex of the APP/PS1 transgenic mice. The expression of retinoic acid receptors (RAR-α, RAR-β, and RAR-γ), retinoid X receptors (RXR-α, RXR-β, and RXR-γ), retinaldehyde dehydrogenases (RALDH1, RALDH2, and RALDH3), and CYP26B1 were determined by real-time quantitative PCR (RT-qPCR). VAD, vitamin A deficiency diet; VAN, vitamin A normal diet. *n* = 3 per group. ^∗^*P* < 0.05, ^∗∗^*P* < 0.01, and n.s., non-significant.

Compared with the VAN group, the transcription of the RARγ, RALDH1, RXRα, RXRβ, and RXRγ genes in the cortex were markedly decreased in the VAD group (*P* < 0.05; Figure 2). Furthermore, VAD decreased the expression of CYP26B1, a catabolizing enzyme that degrades the RA (Woloszynowska-Fraser et al., 2020), in the cortex of APP/PS1 mice (*P* < 0.05; Figure 2). Besides, the transcription of the RARα, RARβ, RALDH2, and RALDH3 genes was reduced in the VAD group compared with the VAN group, but the differences were not statistically significant (n.s.; Figure 2).

### Vitamin A Deficiency Exacerbated β-Amyloid Deposition, Tau Phosphorylation, and Pathological Degeneration

**FIGURE 3:**
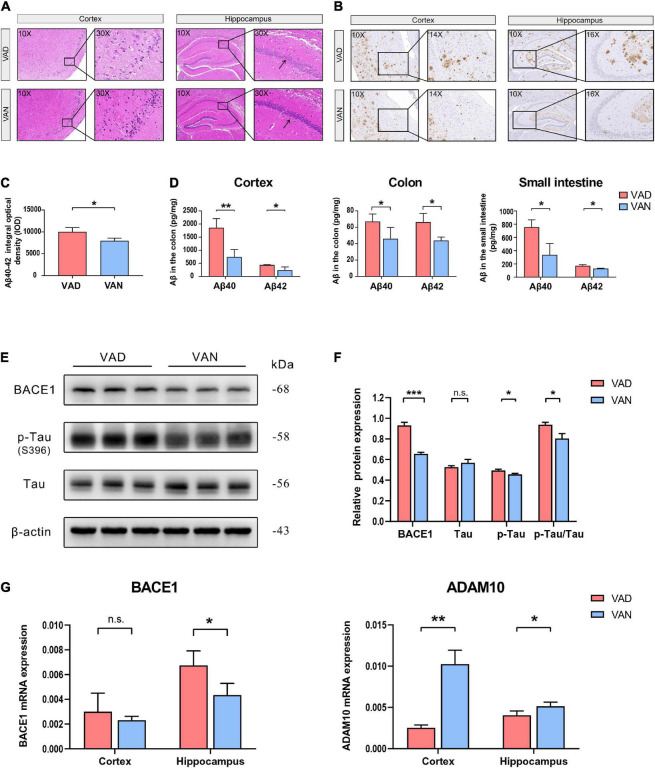
Consumption of the VAD diet for 45 weeks exacerbated the AD-related pathological changes in the cortex, hippocampus, colon, and small intestine of the APP/PS1 transgenic mice. **(A)** Hematoxylin-Eosin (H&E) staining (10× and 30×) in the brain of the APP/PS1 mice. **(B)** Immunohistochemistry (IHC) staining (10×, 14×, and 16×) of Aβ40 and Aβ42 depositions in the brain of the APP/PS1 mice. The primary antibody used for IHC staining could specifically bind both the Aβ40 and Aβ42. **(C)** Quantification of the IHC staining of the Aβ40 and Aβ42 depositions in the cortex of the APP/PS1 mice. **(D)** The Aβ40 and Aβ42 concentrations in the cortex, colon, and small intestine as determined by the Luminex immunoassay. The Luminex immunoassay can detect the concentration of Aβ40 and Aβ42, respectively. **(E)** Western blot (WB) images showing the level of BACE1, Tau proteins, and the phosphorylated Tau (p-Tau, S396) protein in the cortex of the APP/PS1 mice. **(F)** Quantification of the WB result showing the relative level of BACE1, Tau, p-Tau, and the ratio of p-Tau toTau. **(G)** The RT-qPCR result showing the mRNA level of the BACE1 and ADAM10 genes in the cortex and hippocampus of the APP/PS1 mice. VAD, vitamin A deficiency diet; VAN, vitamin A normal diet. *n* = 3 per group. ^∗^*P* < 0.05, ^∗∗^*P* < 0.01, ^∗∗∗^*P* < 0.001, and n.s., non-significant.

From the results of H&E staining, it can be seen that there may be some histopathological changes in the cortex and hippocampus of VAD-diet-fed mice, such as sparse and disordered neuron arrangement (Figure 3A).

To assess the effect of VA deprivation on the Aβ depositions, IHC staining of the brain tissue with the specific Aβ antibody was carried out, and it showed a remarkable increment of Aβ plaque burden in the brain of the VAD mice as compared with the VAN group (Figure 3B). Moreover, our IHC relative quantitative results advocated that the IOD value of Aβ was greatly increased in the VAD mice compared with that in the VAN mice (*P* < 0.05; Figure 3C).

Consistent with the IHC staining, the Luminex results showed that Aβ40 and Aβ42 in the cortex were remarkably increased in the VAD group compared with the VAN group (*P* < 0.05; Figure 3D). Intriguingly, we found that the Aβ burden of the colon and small intestine increased after 45-week VAD-diet consumption (*P* < 0.05; Figure 3D).

As shown in Figures 3E,F, the expression of the BACE1 and p-Tau in the cortex of mice in the VAD group were notably higher than those in the VAN group (*P* < 0.05). Additionally, the ratio of p-Tau to Tau in the VAD group was higher than that in the VAN group (*P* < 0.05).

The mRNA expression of BACE1 in both the cortex and hippocampus of the VAD group was higher than that of the VAN group, but the difference was statistically significant only in the hippocampus (Figure 3G). Moreover, VAD decreased the expression of ADAM10, an enzyme that decreases the formation of Aβ from APP(Shudo et al., 2009), in the cortex and hippocampus (*P* < 0.05; Figure 3G).

Given the findings described above, VAD intensified Aβ generation, Tau phosphorylation, and pathological degeneration in the AD model mice.

### Vitamin A Deficiency Aggravated the Learning and Memory Deficits in Amyloid Precursor Protein/Presenilin 1 Transgenic Mice

**FIGURE 4:**
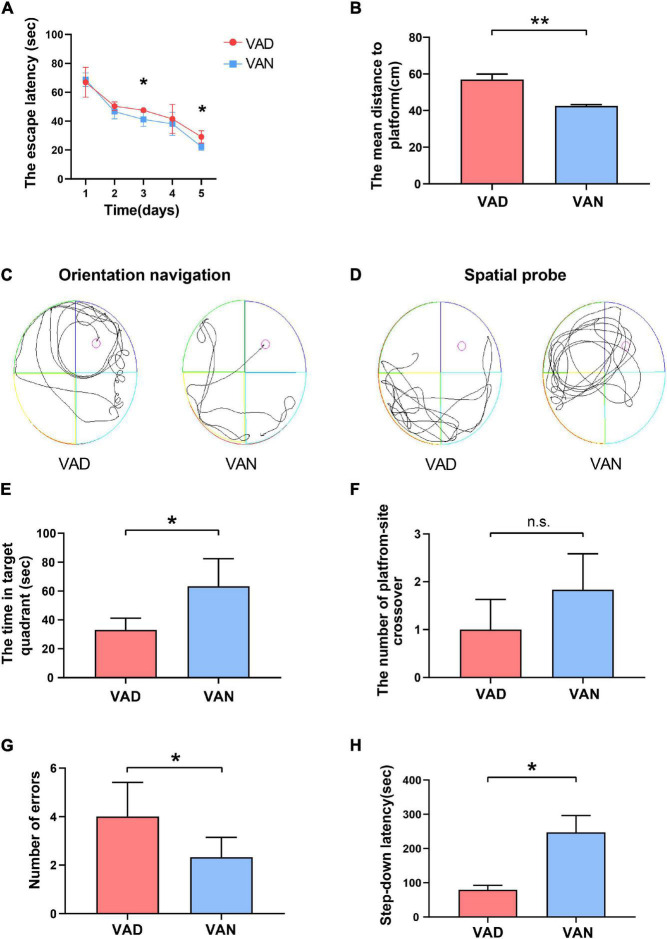
Consumption of the VAD diet for 45 weeks aggravated the learning and memory deficits of the APP/PS1 transgenic mice. **(A)** The escape latency (time to reach the escape platform over days) in the Morris water maze (MWM) test. **(B)** The escape distance in the MWM test. **(C)** The orientation navigation path in the MWM test. **(D)** The spatial probe path in the MWM test. **(E)** The target-quadrant abidance was measured in the MWM test. **(F)** The crossing-target number was measured in the MWM test. **(G)** The error frequency to step down was measured in the step-down passive avoidance (SDPA) test. **(H)** The latency of the step-down response was measured in the SDPA test. VAD, vitamin A deficiency diet; VAN, vitamin A normal diet. *n* = 10 per group. ^∗^*P* < 0.05, ^∗∗^*P* < 0.01, and n.s., non-significant.

To examine the effect of VAD on spatial learning and memory ability, the MWM test was performed. During the orientation navigation, the escape latency to the target platform during the 5 consecutive days is illustrated in Figure 4A. On the third and the fifth day of the test, the escape latency of the VAD group was prolonged as compared with the VAN group (*P* < 0.05). Moreover, the VAD mice traveled longer distances than the VAN mice to reach the platform (*P* < 0.01; Figure 4B,C). In the spatial probe test, compared with the VAN mice, the VAD mice traveled more randomly in the tank without knowing the target location, which demonstrates poor performance in the spatial learning and memory tasks (Figure 4D). Furthermore, the VAD mice spent less time on the target quadrants and crossed the target platform less frequently than the VAN group, but only the difference in the time of target quadrants was statistically significant (Figures 4E,F).

We conducted the SDPA tests to observe the state-dependent learning and memory, 1 day after completing the MWM tests. As illustrated in Figure 4G, the average number of errors in the VAD mice was remarkably higher than in the VAN mice (*P <* 0.05). Moreover, compared with the VAN group, the latency to step-down was notably shortened in the VAD group (*P* < 0.05; Figure 4H).

Both the MWM and SDPA test indicated that VAD could aggravate the learning and memory impairment, thus exacerbating the cognitive deficits in the APP/PS1 transgenic mice.

### Vitamin A Deficiency Altered the Structure and Function of the Gut Microbiota in Amyloid Precursor Protein/Presenilin 1 Transgenic Mice

**FIGURE 5:**
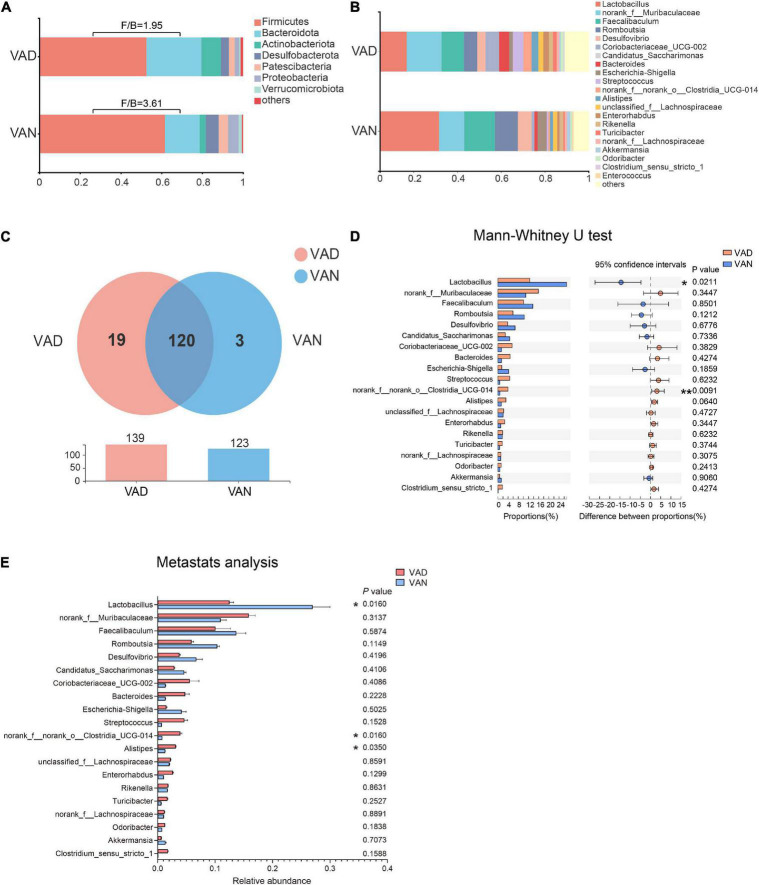
Consumption of the VAD diet for 45 weeks altered the gut microbiota of APP/PS1 transgenic mice. **(A)** The fecal microbiota composition at the phylum level. **(B)** The fecal microbiota composition at the genus level. **(C)** Venn diagram illustrated the overlap and number of the genus identified in the fecal samples obtained from the VAD- and VAN-fed mice. **(D)** Comparison of the differences of the top 20 genera identified in the fecal samples using the Mann–Whitney U test. **(E)** Comparison of the differences of the top 20 genera identified in the fecal samples using Metastats analysis. F/B, the *Firmicutes*/*Bacteroidetes* ratio; VAD, vitamin A deficiency diet; VAN, vitamin A normal diet. *n* = 10 per group. ^∗^*P* < 0.05 and ^∗∗^*P* < 0.01.

**FIGURE 6:**
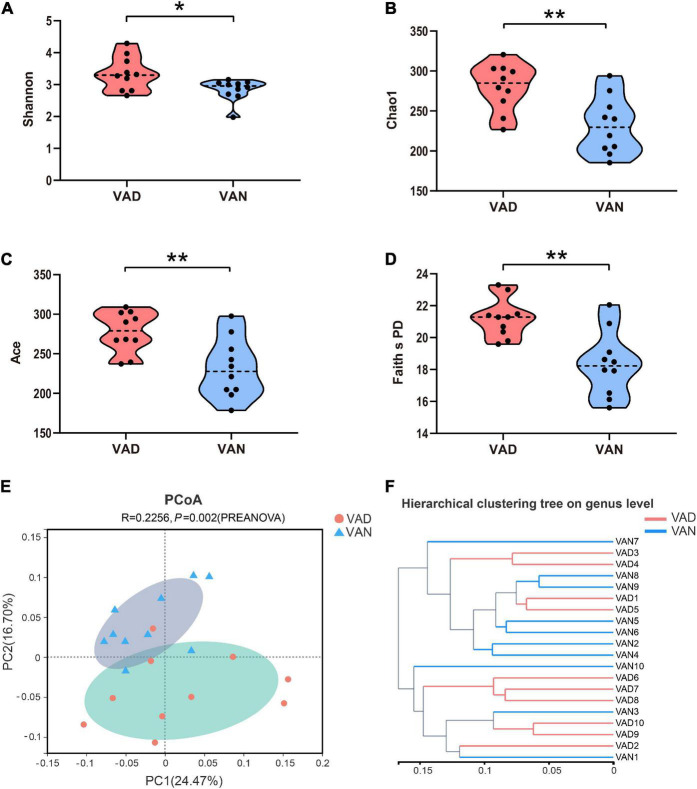
Alpha- and Beta-diversity indices of the fecal microbiota of the APP/PS1 transgenic mice after consumption of the VAD- or VAN-diet for 45 weeks. **(A)** Shannon index. **(B)** Chao1 index. **(C)** Ace index. **(D)** Faith’s phylogenetic diversity index. **(E)** Principal coordinates analysis (PCoA) plot based on the unweighted UniFrac distance. A red dot denotes samples from the VAD group, a cyan triangle denotes samples from the VAN group. **(F)** Hierarchical clustering of the individual samples based on the unweighted UniFrac distance. VAD, vitamin A deficiency diet; VAN, vitamin A normal diet. *n* = 10 per group. ^∗^*P* < 0.05 and ^∗∗^*P* < 0.01.

To evaluate the effect of VAD on the intestinal microbiota, sequencing of the V3–V4 regions of the bacterial 16S rRNA was performed on the fecal samples. In the phylum level, the *Firmicutes* (F)/*Bacteroidetes* (B) ratio of the VAD group and the VAN group were 1.95 and 3.61, respectively, as displayed in Figure 5A. Community abundance at class, order, and family level are shown in Supplementary Figure 3. Of note, the VAD mice exhibited an enrichment of some potential pro-AD microorganisms, such as *Clostridia_UCG-014*, and a reduction in the abundance of other potential anti-AD microorganisms, such as *Lactobacillus*, compared with the VAN mice (Figures 5B,D,E; *P* < 0.05). The data of Mann–Whitney U test and Metastats analysis for all genera are shown in Supplementary Tables 1, 2. Venn diagram at the genus level (Figure 5C) revealed that 120 genera were shared by the VAD and the VAN group, but mice on the VAD-diet have more genus than those on the VAN-diet. After VAD intervention, three genera (*Formosa, Rhodobacteraceae*, and *Alloprevotella*) were missing, while 19 new genera (*Sphingomonas, Peptoniphilus, Rodentibacter*, etc.) have appeared (Supplementary Table 3). Interestingly, we found that most of the 19 genera in the VAD diet are Gram-positive microbes, while the three genera in the VAN diet are Gram-negative microbes. As a result, the α-diversity indices (Shannon, Chao1, ACE, and Faith’s PD) in the VAD group were remarkably higher than those in the VAN group (*P* < 0.05; Figures 6A–D). It means that, compared with the VAN-diet, the consumption of the VAD-diet for 45 weeks increased the community evenness and richness in the fecal microbiota of the APP/PS1 mice. The PCoA plot and the hierarchical clustering tree manifested that most of the samples could be separated according to their diets (Figures 6E,F). The dissimilarity of the microbiota between the two groups was further confirmed by the statistical analysis of the differences with the PERMANOVA analysis (*P* = 0.002).

**FIGURE 7:**
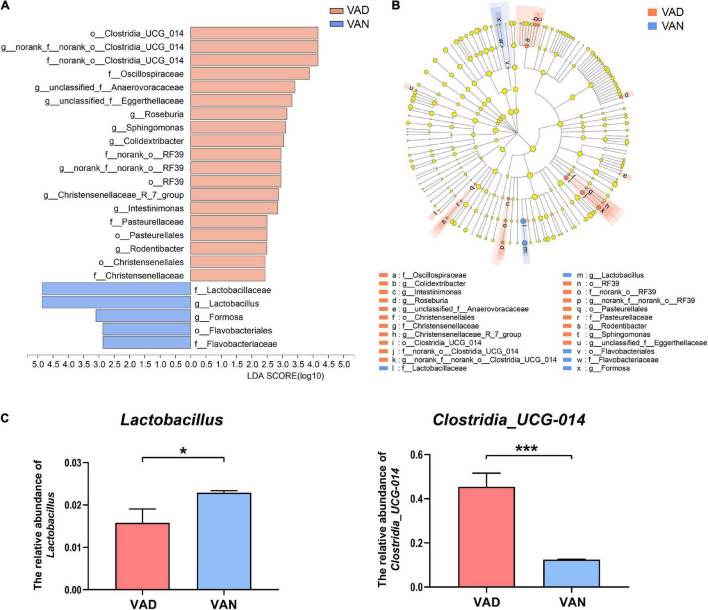
Linear discriminant analysis (LDA) effect size (LEfSe) analysis of the 16S rRNA gene sequencing data and the RT-qPCR verified abundance of *Lactobacillus* and *Clostridia_UCG-014*. **(A)** Histogram of the LDA scores computed for differentially abundant bacterial taxa between the VAD and VAN groups. Only taxa with a LDA score (log_10_) > 2 and *P* < 0.05 are listed. **(B)** Cladogram showing the impact of the diet on the taxonomic distribution of the bacteria. A total of 23 differentially abundant bacterial taxa were detected. Of those, seven bacterial taxa were significantly overrepresented in the VAN group (blue) and 15 bacterial taxa were overrepresented in the VAD group (red). **(C)** The RT-qPCR validation of the changes in the relative abundance of *Lactobacillus* and *Clostridia_UCG-014* by using their specific primers. VAD, vitamin A deficiency diet; VAN, vitamin A normal diet. *n* = 10 per group. ^∗^*P* < 0.05, and ^∗∗∗^*P* < 0.001.

The LEfSe algorithm makes a supervised comparison of the microbiota to elucidate variant taxa at different taxonomic levels between the two groups. The LEfSe analysis demonstrated that, compared with the VAN group, the intestinal microbiota of the VAD group was different in 12 bacterial genera, seven bacterial families, and five bacterial orders (Figures 7A,B). Particularly, the potentially beneficial bacteria *Lactobacillus* in the VAD group was significantly lower than that in the VAN mice, while the potentially harmful bacteria *Clostridia_UCG-014* in the VAD group was greatly higher than that in the VAN mice, which was consistent with the Mann–Whitney U test and Metastats analysis in Figures 5D,E. Moreover, the reduction in *Lactobacillus* and the increment in *Clostridia_UCG-014* were further verified by RT-qPCR using their respective taxa-specific primers (*P* < 0.05; Figure 7C).

**FIGURE 8:**
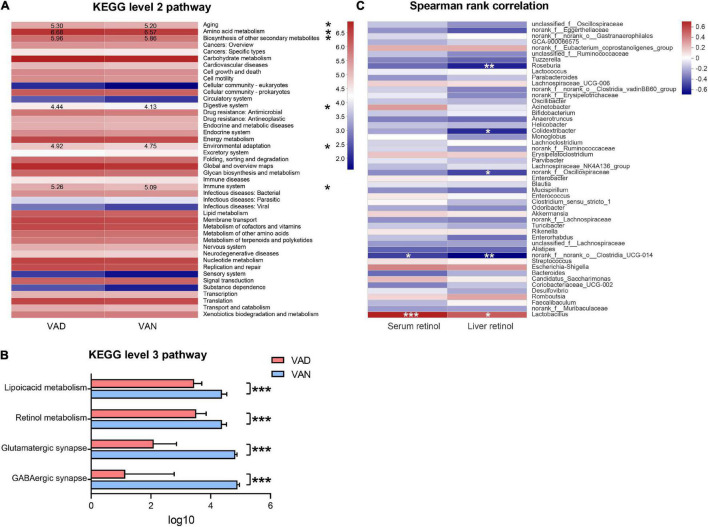
Predicted function of the gut microbiota and its correlation with the liver retinol and serum retinol. **(A)** Comparisons of the VAD- and VAN-diet impact on the predicted functions of the gut microbiota at the Kyoto Encyclopedia of Gene and Genomes (KEGG) level 2 by using the PICRUSt analysis. The number indicates the logarithmic value of enrichment to the pathways. **(B)** Significantly different functions of the gut microbiota at KEGG level 3 between the VAN and VAD group as predicted by the PICRUSt analysis (partial). **(C)** Correlation of the liver retinol and the serum retinol with the relative abundance of the top 50 bacterial genera in all mice. The red color represents a positive correlation. Blue color represents a negative correlation. VAD, vitamin A deficiency diet; VAN, vitamin A normal diet. *n* = 10 per group. ^∗^*P* < 0.05, ^∗∗^*P* < 0.01, and ^∗∗∗^*P* < 0.001.

We used the PICRUSt analysis to predict the functional changes in the metagenome of the present experiment. In the KEGG pathway prediction results, quite a few metabolic pathways were altered in the VAD mice. Interestingly, the VAD mice significantly enriched the pathways related to aging, amino acid metabolism, biosynthesis of other secondary metabolites, digestive system, environmental adaptation, and immune system at KEGG level 2, while lowered those involved in the GABAergic synapse, glutamatergic synapse, retinol metabolism, and lipoic acid metabolism at KEGG level 3 (*P <* 0.05; Figures 8A,B and Supplementary Table 4). To identify whether VAD diet-driven intestinal microbiota changes are related to the VA concentration or not, we performed correlation analyses between these parameters. Spearman’s rank correlation indicated that both the liver retinol and serum retinol were positively correlated with the abundance of *Lactobacillus* (*r* = 0.51, *P <* 0.05; *r* = 0.70, *P <* 0.001; Figure 8C) and negatively correlated with the abundance of *Clostridia_UCG-014* (*r* = − 0.66, *P <* 0.01; *r* = −0.49, *P <* 0.05; Figure 8C and Supplementary Table 5).

### Vitamin A Deficiency Reduced the Expression of Brain-Derived Neurotrophic Factor and γ-Aminobutyric Acid Receptors in the Brain of the Amyloid Precursor Protein/Presenilin 1 Mice

**FIGURE 9:**
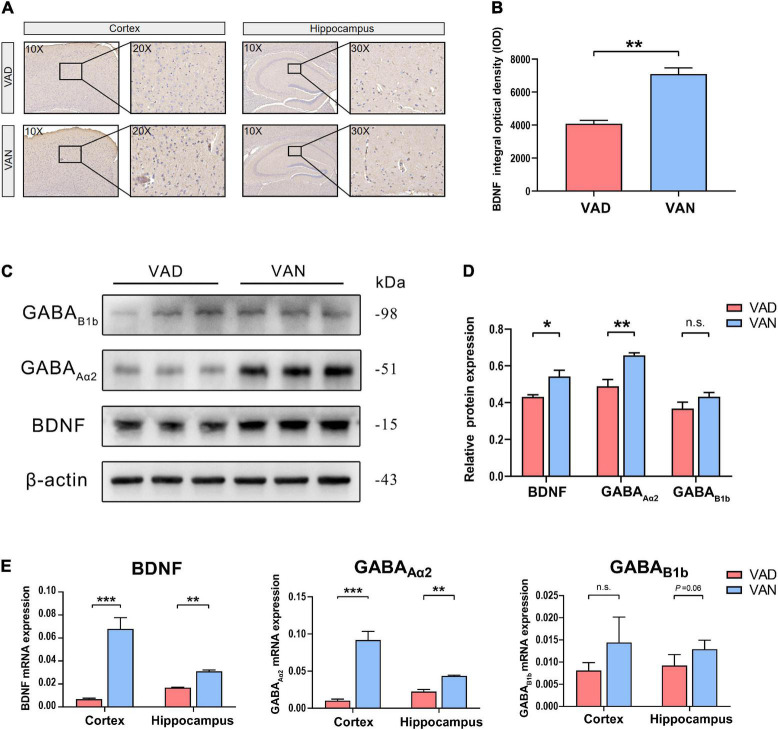
Consumption of the VAD diet for 45 weeks reduced the expression of brain-derived neurotrophic factor (BDNF) and the γ-aminobutyric acid (GABA) receptors in the cortex and hippocampus of the APP/PS1 transgenic mice. **(A)** The IHC images (10×, 20×, and 30×) showing the staining of the BDNF in the brain. **(B)** Quantification of the BDNF level in the brain of the mice in the IHC assay. **(C)** The WB images showing the expression of BDNF, GABA_Aα2_, and GABA_B1b_ in the cortex of the APP/PS1 mice fed with either VAD or VAN. **(D)** The BDNF, GABA_Aα2_, and GABA_B1b_ were quantitated according to the band density and normalized against the levels of β-actin. **(E)** RT-qPCR analysis of the expression of BDNF, GABA_Aα2_, and GABA_B1b_ genes in the cortex and hippocampus of the APP/PS1 mice. VAD, vitamin A deficiency diet; VAN, vitamin A normal diet. *n* = 3 per group. ^∗^*P* < 0.05, ^∗∗^*P* < 0.01, ^∗∗∗^*P* < 0.001, and n.s., non-significant.

To assess the effect of long-term VAD on the brain BDNF expression and secretion, we measured the BDNF in the cortex and hippocampus with IHC, western blot (WB), and RT-qPCR, respectively. As shown in Figure 9, compared with the VAN mice, the BDNF gene and the protein expression were markedly downregulated in the VAD-diet APP/PS1 transgenic mice (*P <* 0.05).

To verify the results of functional metagenomic prediction about the GABAergic synapse, Western blotting and RT-qPCR were employed to determine the expression of the GABA_Aα2_ and GABA_B1b_ in the cortex and hippocampus of the APP/PS1 mice. As shown in Figures 9C,D, compared with the VAN group, the protein expression of GABA_Aα2_ and GABA_B1b_ were downregulated in the cortex of the VAD-diet-fed mice, but only the GABA_Aα2_ was significantly changed. Moreover, the RT-qPCR results demonstrated that the transcription of GABA_Aα2_ in the cortex and hippocampus of APP/PS1 mice in the VAD group was notably lower than those in the VAN group (*P* < 0.05; Figure 9E), which was consistent with the WB results.

## Discussion

Vitamin A deficiency in older age is considered a modifiable risk factor for AD and other cognitive disorders (Woloszynowska-Fraser et al., 2020). However, the prevalence of VAD in developing countries, particularly among older persons with AD, demonstrates the importance of ensuring adequate VA intake for seniors. Diet-derived VA, which are mainly stored in the liver, is released in a homeostatically controlled way to provide a constant source of RA to cells of the body, including the brain cells (Pardridge et al., 1985). Retinol from the liver can also pass through the blood–brain barrier (BBB) by binding to the retinol-binding protein 1 on the choroid plexus and vascular walls of the brain (Woloszynowska-Fraser et al., 2020). After entering the BBB, retinol is sequentially transformed to retinal and RA by the retinol dehydrogenase and retinaldehyde dehydrogenase (RALDH), respectively. VAD in rodents impairs both long-term potentiation and long-term depression in the brain, and the degree of their disruption correlates with the level of VA deprivation (Misner et al., 2001). VA deficiency may further aggravate the decline in RA signaling (Zeng et al., 2017a) because such conditions lead to a decrease in the expression of RARs and RXRs. In addition, the studies in RARβ/RXRγ knockout mice have shown that lack of these receptors leads to decreased long-term declarative (conscious) memory and cognitive flexibility (Mingaud et al., 2008). This evidence is in line with our results that long-term VA deprivation resulted in a significantly decreased serum and liver retinol level and reduced the mRNA expression of the VA-related receptors (RARγ, RXRα, RXRβ, and RXRγ) and VA-related enzyme (RALDH1 and CYP26B1) in the cortex of the APP/PS1 mice.

Accumulating studies illustrate that Aβ deposition and Tau hyperphosphorylation are tightly related to the cognitive deficits and participate in the pathological mechanism of AD (Ramos-Rodriguez et al., 2013). Through IHC staining and Luminex immunoassay in the AD model mice, our research shows that the Aβ plaque load significantly increased after VA deprivation. Interestingly, we found that the Aβ levels were increased in the small intestine and colon of the mice after VA deprivation. In addition, previous studies have illustrated that enteric Aβ may translocate to the brain *via* axonal transportation in the vagal nerves (Sun et al., 2020). Although VAD did not affect the total Tau levels, it significantly increased the phosphorylated Tau protein, suggesting that VA deprivation facilitated Tau hyperphosphorylation in the cortex of mice. Additionally, the increased expression of BACE1 indicated that VAD might lead to Aβ plaque accumulation by dysregulating BACE1 expression, which is consistent with previous studies (Wang et al., 2015; Zeng et al., 2017b). In addition to the increase in Aβ, p-Tau, and BACE1, the decreased mRNA expression of ADAM10 suggested that VA deprivation may facilitate AD pathogenesis. These pathological manifestations are consistent with the behavioral results of the MWM test and SDPA test in our study, which are shown as aggravated learning and memory deficits in VAD mice.

Interestingly, our results demonstrate that VA deprivation decreased the mRNA expression of RALDH1 and CYP26B1, suggesting that VA deprivation may lead to a decrease in the synthesis and decomposition of RA in the brain. However, the effect of VAD on RA production and metabolism needs further study to verify.

Considering that there are amounts of undefined soluble amyloid protein secreted by symbionts, the intestinal microbiome may play an essential role in the pathogenesis of neurodegenerative diseases characterized by amyloidogenic features, such as AD (Zhao and Lukiw, 2015). The contribution of the gut microbiota to the formation and dissemination of amyloid in the elderly has become more critical because their gastrointestinal epithelial cells and the BBB have become more permeable to small molecules. Moreover, Shen et al. (2017) reported that the cognitive deficits of APP/PS1 mice are related to the specific gut microbiome states. Although individuals have different gut microbiota, it mainly contains members of four phyla (*Firmicutes*, *Bacteroidete*s, *Actinobacteria*, and *Proteobacteria*), among which *Firmicute*s and *Bacteroidetes* account for the largest proportion (Gerard, 2016). As a simple indicator of intestinal microbiota status, the *Firmicute*s/*Bacteroidetes* (F/B) ratio increased with the age before adulthood (10.9) and then decreased with age (0.6) (Doifode et al., 2021). Vogt et al. (2017) demonstrated that the F/B ratio decreased in the patients with AD. Our results also demonstrated that the F/B ratio decreased in the VAD-diet mice, implying that the intestinal homeostasis was disrupted. The Venn diagram manifested that VAD led to more new genera in the intestinal microbiota, but most were not probiotics. The α-diversity is primarily associated with two factors. One is the number of species: richness; the other is diversity, the evenness of individual distribution in the community (Koh, 2018). Interestingly, the α-diversity increased in the VAD mice, which indicated that either there were more genera in the intestines of the VAD mice or there are more similar distribution of abundances of genera in VAD mice. But that does not mean it is in a better intestinal microbial homeostasis, as it may be caused by the increase or appearance of harmful bacteria, such as the 19 unique bacterial genera in the VAD mice (Supplementary Table 3).

The β-diversity uses the evolutionary relationship and abundance information of the sequences of each sample to estimate the relative distance between the samples to reflect significant differences in the microbial communities between the groups (Soininen et al., 2007). The PCoA plot indicated that the composition, species, and differentiation of gut microbiota altered significantly after long-term VAD.

The Mann–Whitney U test, LEfSe analysis, and RT-PCR validation showed that the genus *Lactobacillus* decreased significantly after long-term VAD (*P <* 0.05). In addition, the Spearman’s rank correlation analysis showed that the liver and serum retinol level was positively associated with the abundance of *Lactobacillus* (*r* = 0.51, *P <* 0.05; *r* = 0.70, *P <* 0.001). *Lactobacillus* can enhance the intestinal mucosal barrier function by increasing the permeability resistance of HT-29 cells and Caco-2 cells, along with the increase of the phosphorylation level of tight junction proteins (Resta-Lenert and Barrett, 2003). It has been demonstrated that *Lactobacillus* can induce the expression of mucin in the colonic epithelial cells, which forms a protective barrier between the body and the external environment (Caballero-Franco et al., 2007). Moreover, previous studies have demonstrated that *Lactobacillus* could stimulate the gut-brain axis and upregulate the expression of BDNF (Ranuh et al., 2019). The study by Buchman et al. (2016) highlights the brain BDNF as a potential substantial contributor to slowing cognitive deterioration in the elderly, particularly in the context of advancing AD neuropathology. Accumulating studies have shown that BDNF levels decreased in both the brain and serum of the patients with AD (Carlino et al., 2013). In addition, Beeri and Sonnen (2016) argued that the brain BDNF expression could be regarded as a biomarker for the cognitive improvement against the AD pathological progression. In neurons, BDNF mainly binds to the tyrosine kinase receptor B (TrkB) to activate the intracellular signaling pathways, such as the PI3K-Akt signaling pathway, Ras/Raf-MEK-ERK signaling pathway, and PLC-PKC signaling pathway, thereby improving the viability and regeneration of the neurons (Hang et al., 2021) and increasing the synaptic plasticity and the learning and memory function of the brain (Zhang et al., 2012). On the contrary, the BDNF-devoid neurons often manifest neurofibrillary tangles, a characteristic of AD, which was absent in the densely BDNF-labeled neurons (Murer et al., 1999). Furthermore, Nigam et al. (2017) demonstrated that BDNF reduces the Aβ production by enhancing α-secretase processing of the APP. Therefore, BDNF may be an important mediator of the aggravated cognitive deficits after long-term VAD.

Meanwhile, we observed that the abundance of *Clostridia_UCG-014* increased significantly in the VAD mice. Similar to the results of *Lactobacillus*, this difference was also verified by Mann–Whitney U-test, Metastats analysis, LEfSe analysis, Spearman’s rank correlation analysis, and RT-qPCR. *Clostridia_UCG-014* is an obligately anaerobic bacteria, which is common in soil and the mammalian intestine (Suen et al., 2007). Previous studies have shown that the increased abundance of *Clostridia_UCG-014* is related to elevated fasting blood glucose (Zhao et al., 2021) and colitis (Liu et al., 2021). *Clostridia*-related diseases mainly occur when the spores enter the body, colonize the host, germinate and generate toxins (Mallozzi et al., 2010). Thus, the increase in *Clostridia_UCG-014* after long-term VAD may cause more serious damage to the health of the patients with AD.

Functional interpretation of the fecal metagenomes by PICRUSt revealed that the pathways, such as amino acid metabolism, GABAergic synapse, glutamatergic synapse, retinol metabolism, and lipoic acid metabolism displayed remarkable alterations after long-term VAD. The decrease in the retinol metabolism pathway is consistent with the decrease of the retinol level in the liver and the serum and the decrease of VA-related receptors in the brain. An AD microbiome cohort (Liu et al., 2019) revealed that decreased amino acid metabolism was found in the patients with cognitive impairment. In addition, the function and structure of glutamatergic synapses are affected by Aβ in the early stage of AD (Marcello et al., 2012). Holmquist et al. (2007) found that lipoic acid could be regarded as a neuroprotective and anti-inflammatory treatment for patients with AD. Besides, the GABAergic synapses play a neuroprotective role in the process of Aβ-induced neurotoxicity by releasing GABA (Cisternas et al., 2020). More interestingly, Bravo et al. (2011) highlighted that *Lactobacillus* could upregulate the GABA receptors in the hippocampus and cortex through the vagus nerve, which is consistent with our RT-qPCR and WB results about the GABA_Aα2_ and GABA_B1b_ receptors. Moreover, BDNF can promote the synaptic formation and maturation of the GABAergic synapses (Huang et al., 1999), and the excitatory action of GABA has been shown to activate the BDNF expression (Fukuchi et al., 2014). Therefore, we hypothesize that intestinal microbiota, especially *Lactobacillus*, may play an important role in the reduction of the BDNF and GABA receptors in the brain after long-term VAD in AD individuals, thereby increasing the production of Aβ and Tau hyperphosphorylation, and ultimately affecting the development of the cognitive dysfunction. Although the above hypothesis can be proposed from the results of our study, we do not know to what extent the AD pathological and behavioral impair can be attributed to the reduced BDNF and GABA receptors by the VAD-induced microbiota disruption. The present study is only a preliminary observation, but we provide a new perspective on the gut-brain axis that VAD exacerbates the gut microbiota dysbiosis and cognitive deficits, and we will conduct further experiments to verify this hypothesis in the future.

## Conclusion

In summary, the findings of the present study provided valuable facts that long-term VAD exacerbates the gut microbiota dysbiosis and cognitive deficits, highlighting the importance of monitoring the VA levels during senescence and necessitating the timely supplementation of VA in person who is at a higher risk of developing AD. Additionally, we posit that VAD may reduce the expression of GABA receptors and downregulate BDNF in the brain by disturbing intestinal microbiota (especially *Lactobacillus*), thus leading to histological and cognitive impairment in AD. Further studies are warranted to verify the hypothesis and elucidate the mechanisms by which VAD exerts its effect on microecology.

## Data Availability Statement

The datasets presented in this study can be found in online repositories. The names of the repository/repositories and accession number(s) can be found in the article/Supplementary Material.

## Ethics Statement

The animal study was reviewed and approved by the Ethics Committee of Capital Medical University (AEEI-2018-176).

## Author Contributions

P-GL and CY had primary responsibility for the final content and designed the research. B-WC and K-WZ conducted the experiment. B-WC analyzed the data and wrote the manuscript. S-JC revised the manuscript. All authors contributed to the article and approved the submitted version.

## Conflict of Interest

The authors declare that the research was conducted in the absence of any commercial or financial relationships that could be construed as a potential conflict of interest.

## Publisher’s Note

All claims expressed in this article are solely those of the authors and do not necessarily represent those of their affiliated organizations, or those of the publisher, the editors and the reviewers. Any product that may be evaluated in this article, or claim that may be made by its manufacturer, is not guaranteed or endorsed by the publisher.
