# Supplementary material for: Vitamin A Deficiency Exacerbates Gut Microbiota Dysbiosis and Cognitive Deficits in Amyloid Precursor Protein/Presenilin 1 Transgenic Mice
Source: Front Aging Neurosci. 2021 Nov 1;13:753351. doi: 10.3389/fnagi.2021.753351 (PMC8591312; doi:10.3389/fnagi.2021.753351)
Supplement: Supplementary file 6 [file Data_Sheet_1.DOCX]

Supplementary Material

## Supplementary Figure 1





## **Supplementary Figure 1.** Representative HPLC traces of retinol in liver. (A) Representative HPLC trace of retinol in the liver of VAD-diet-fed mice. (B) Representative HPLC trace of retinol in the liver of VAN-diet-fed mice. VAD, vitamin A deficiency diet; VAN, vitamin A normal diet. n = 10 per group.

## Supplementary Figure 2





## **Supplementary Figure 2.** Representative HPLC traces of retinol in serum. (A) Representative HPLC trace of retinol in the serum of VAD-diet-fed mice. (B) Representative HPLC trace of retinol in the serum of VAN-diet-fed mice. VAD, vitamin A deficiency diet; VAN, vitamin A normal diet. n = 10 per group.

## Supplementary Figure 3


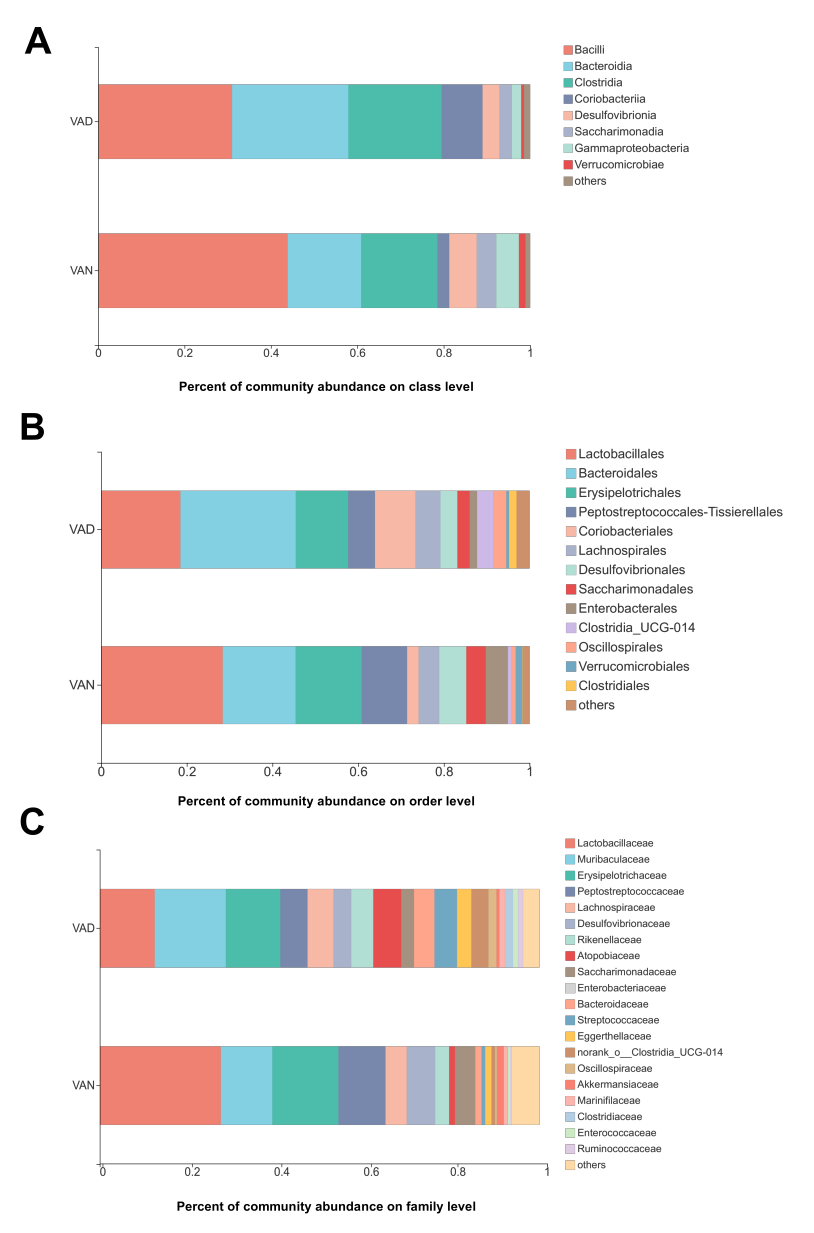


**Supplementary Figure 3.** Consumption of the VAD diet for 45 weeks altered the microbiota composition of APP/PS1 transgenic mice. (A) Fecal microbiota composition at the class level. (B) Fecal microbiota composition at the order level. (C) Fecal microbiota composition at the family level. VAD, vitamin A deficiency diet; VAN, vitamin A normal diet. n = 10 per group.
